# TC-PTP Dephosphorylates the Guanine Nucleotide Exchange Factor C3G (RapGEF1) and Negatively Regulates Differentiation of Human Neuroblastoma Cells

**DOI:** 10.1371/journal.pone.0023681

**Published:** 2011-08-18

**Authors:** Aninda Mitra, Srinivasan Kalayarasan, Vijay Gupta, Vegesna Radha

**Affiliations:** Centre for Cellular and Molecular Biology, Council of Scientific and Industrial Research (CSIR), Hyderabad, Andhra Pradesh, India; University of São Paulo, Brazil

## Abstract

The guanine nucleotide exchange factor, C3G (RapGEF1), functions in multiple signaling pathways involved in cell adhesion, proliferation, apoptosis and actin reorganization. C3G is regulated by tyrosine phosphorylation on Y504, known to be mediated by c-Abl and Src family kinases. In the present study we explored the possibility of cellular phospho-C3G (pC3G) being a substrate of the intracellular T-cell protein tyrosine phosphatase TC-PTP (PTPN2) using the human neuroblastoma cell line, IMR-32. *In vivo* and *in vitro* binding assays demonstrated interaction between C3G and TC-PTP. Interaction is mediated through the Crk-binding region of C3G and C-terminal noncatalytic residues of TC-PTP. C3G interacted better with a substrate trap mutant of TC48 and this complex formation was inhibited by vanadate. Endogenous pC3G colocalized with catalytically inactive mutant TC48 in the Golgi. Expression of TC48 abrogated pervanadate and c-Src induced phosphorylation of C3G without affecting total cellular phospho-tyrosine. Insulin-like growth factor treatment of c-Src expressing cells resulted in dephosphorylation of C3G dependent on the activity of endogenous TC48. TC48 expression inhibited forskolin induced tyrosine phosphorylation of C3G and neurite outgrowth in IMR-32 cells. Our results identify a novel Golgi localized substrate of TC48 and delineate a role for TC48 in dephosphorylation of substrates required during differentiation of human neuroblastoma cells.

## Introduction

Signals initiated by transmembrane receptors at the cell surface are transmitted through the activity of guanine nucleotide exchange factors (GEFs) responsible for activation of small GTPases. Through their action on multiple effector molecules, GTPases enable regulation of diverse cellular functions like proliferation, actin reorganization, adhesion, motility, apoptosis and differentiation. The ubiquitously expressed GEF, C3G (RapGEF1, GRF2) functions in signaling pathways initiated by growth factors, integrins, T and B-cell receptors, cytokines, mechanical force etc and is known to target Rap1, 2, R-Ras and TC-10 [1]–[8]. C3G plays a role in regulating cell proliferation, apoptosis, actin reorganization, neuronal differentiation, and is essential during embryonic development [9]–[14].

C3G is a 140 kDa protein that primarily possesses a catalytic domain at the extreme C-terminus responsible for guanine nucleotide exchange and a central region comprising of multiple proline-rich sequences involved in protein interaction [15], [16]. SH3 domain containing molecules like Crk, Hck, p130 Cas and c-Abl have been shown to interact with C3G through this domain [10], [11], [15]–[17]. Within this domain are also present several tyrosine residues, and Y504 is targeted by Src family kinases (SFKs) and c-Abl [10], [18], [19]. The N-terminal domain of C3G is poorly defined except for sequences responsible for interaction with E-cadherin [20]. Catalytic activity of C3G is regulated through Y504 phosphorylation and membrane targeting [21], [22]. C3G suppresses malignant transformation independent of its catalytic activity [23]. In signaling pathways, C3G therefore has functions dependent on both its catalytic activity and interaction domain.

Knockout mice lacking C3G are embryonic lethal and show defects in multiple systems like vascular maturation and neural cortical development [13], [24], [25]. We have demonstrated that C3G signals to actin reorganization and is required for differentiation of human neuroblastoma cells [12]. Stimulation of ALK, a receptor tyrosine kinase, results in tyrosine phosphorylation of C3G and neurite growth in PC12 cells [26]. Cells differentiated by neurotrophin treatment show enhanced C3G protein levels and Src family kinase dependent phosphorylation of C3G on Y504 [12]. Phospho-C3G (pC3G) under these conditions localizes predominantly at the Golgi. Since phospho-tyrosine dependent signaling is also under the control of tyrosine phosphatases, we wished to identify enzymes that regulate C3G phosphorylation and its downstream effector functions.

The T-cell protein tyrosine phosphatase (TC-PTP) is an intracellular PTPase expressed as two alternately spliced isoforms TC48 and TC45, which differ only in their C-termini [27], [28]. TC48 is localized to the nuclear membrane, endoplasmic reticulum and Golgi [27]–[29]. p23 and p25, proteins of a family of cargo receptors were identified as specific interacting partners of TC48 and enable its dynamic exchange between ER and Golgi compartment [29]. TC45 is present in the nucleus and is known to exit the nucleus in response to some types of stress and growth factor stimulation [30]–[34]. TC-PTP deficient mice show defects in development of the hematopoietic system and in inflammatory responses [35]. TC-PTP is ubiquitously expressed and has functions in insulin signaling [36]. Its role in other cells and tissues is yet to be determined. Identification of TC-PTP substrates has been important in understanding its physiological functions. While a large number of cellular substrates have been identified for TC45, very few TC48 substrates are known [37]. Since subcellular localization plays an important role in determining substrate specificity [38], in this study, we investigated the ability of TC-PTP isoforms to dephosphorylate cellular C3G in human neuroblastoma cells. Our results identify C3G as a novel interacting partner and substrate of TC48. We also describe a functional significance for TC48 mediated dephosphorylation in differentiation of human neuroblastoma cells.

## Materials and Methods

### Cell lines and transfections

IMR-32, human neuroblastoma cells, HEK293, human embryonic kidney cells and Cos-1, monkey kidney epithelial cells, obtained from ATCC were maintained in DMEM containing 10% FCS at 37°C in a humidified incubator with 5% CO_2_. Cells were grown as monolayers in dishes for lysate preparation or on coverslips for immunofluorescence. Transfection of IMR-32 cells and Cos-1 cells were performed as described [12], [18]. Generally 400 ng of DNA was used to transfect cells on coverslips or in 24 well plates and 4 µg was used for cells in 60 mm dishes. Transient transfection was carried out for 30–48 hours. Cells were treated with 50 µM sodium vanadate for 4 hrs or with 50 µM pervanadate for 10 min prior to fixation or lysate preparation. IGF (100 ng/ml) treatment was performed for 4 hrs after subjecting cells to serum starvation by incubating them in serum-free medium for 8–10 hrs. Forskolin treatment for differentiation of IMR-32 cells was carried out as described earlier [12].

### Expression vectors and antibodies

Plasmids for expression of full length C3G, Y504FC3G, GST-CBR, GFP-c-Src, c-Src, GFP-TC48, GFP-TC45, GFP-TC48-C-66, and GFP-p23 have been described earlier [11], [18], [29]. Y504F C3G is a phosphorylation site mutant. GST-CBR is a recombinant protein with the Crk binding region of C3G fused to GST. GFP-TC45-C38 is a deletion construct of TC45 containing C-terminal residues 350-387 cloned in pEGFP vector. Mutant TC48 and TC45 were created by substituting aspartic acid at 182 to alanine using site-directed mutagenesis (D182A, mTC48/mTC45). These mutants lack catalytic activity and function as dominant negatives as well as substrate traps [38], [39].

Antibodies against C3G (C-19); pC3G (pY504-C3G), c-Src, Cdk2, p-Tyr and GFP were from Santa Cruz. TC-PTP antibody which recognizes both human isoforms was from Calbiochem. ERGIC-53 antibody was from Axxora, and giantin antibody was from Covance.

#### Immunoprecipitation, *in vitro* binding assays and western blotting

Immunoprecipitations were carried out essentially as described [11], [29]. Lysates prepared from HEK293 cells expressing C3G and GFP-TC-PTP constructs, or mTC48 alone were incubated with equal amounts of GFP antibody or control mouse IgG. Immune complexes were isolated using protein A/G plus agarose beads and subjected to western blotting using C3G and GFP antibodies. Expression of GST-C3G-CBR fusion protein and *in vitro* binding assays were performed as described [11]. Lysates of HEK293 cells expressing various TC-PTP constructs were used for *in vitro* binding. Western blotting was carried out using standard protocols. Conditions for Western blotting of pC3G have been described [10], [18]. About 50 µg of lysates were loaded to visualize the proteins by western blot. Probing using multiple antibodies was carried out after deprobing.

### Indirect immunofluorescence and quantitation of neurite outgrowth

Expression and localization of overexpressed or endogenous protein was determined in cells grown on coverslips. Essentially, cells were fixed in 3.7% formaldehyde in PBS for 10 mins and processed for staining as described [18], [19]. Secondary antibodies coupled to Cy3, Cy5, Alexa-488 or Alexa-633 were used. Sequential staining was carried out to detect the presence of two different proteins. Co-localization was examined by analyzing optical sections in the Z-axis using 63X oil immersion objective of a confocal microscope (Leica). Images were also captured using 40X objective of Zeiss Axioplan 2 microscope fitted with an Apotome.

Effect of TC-PTP on neurite outgrowth (NOG) in IMR-32 cells was determined by transfecting GFP-TC48 or mTC48 plasmids and inducing differentiation by 25 µM forskolin treatment for 72 hrs. NOG was quantitated as described by determining the number of cells with extensions greater than 2 cell diameters among the GFP expressing and non-expressing cells [12]. Data was averaged from a minimum of 3 experiments carried out on duplicate cover slips and represented as mean ± sd. Significance of the data was determined using Students ‘t’ test.

## Results

### C3G interacts with TC48

**Figure 1:**
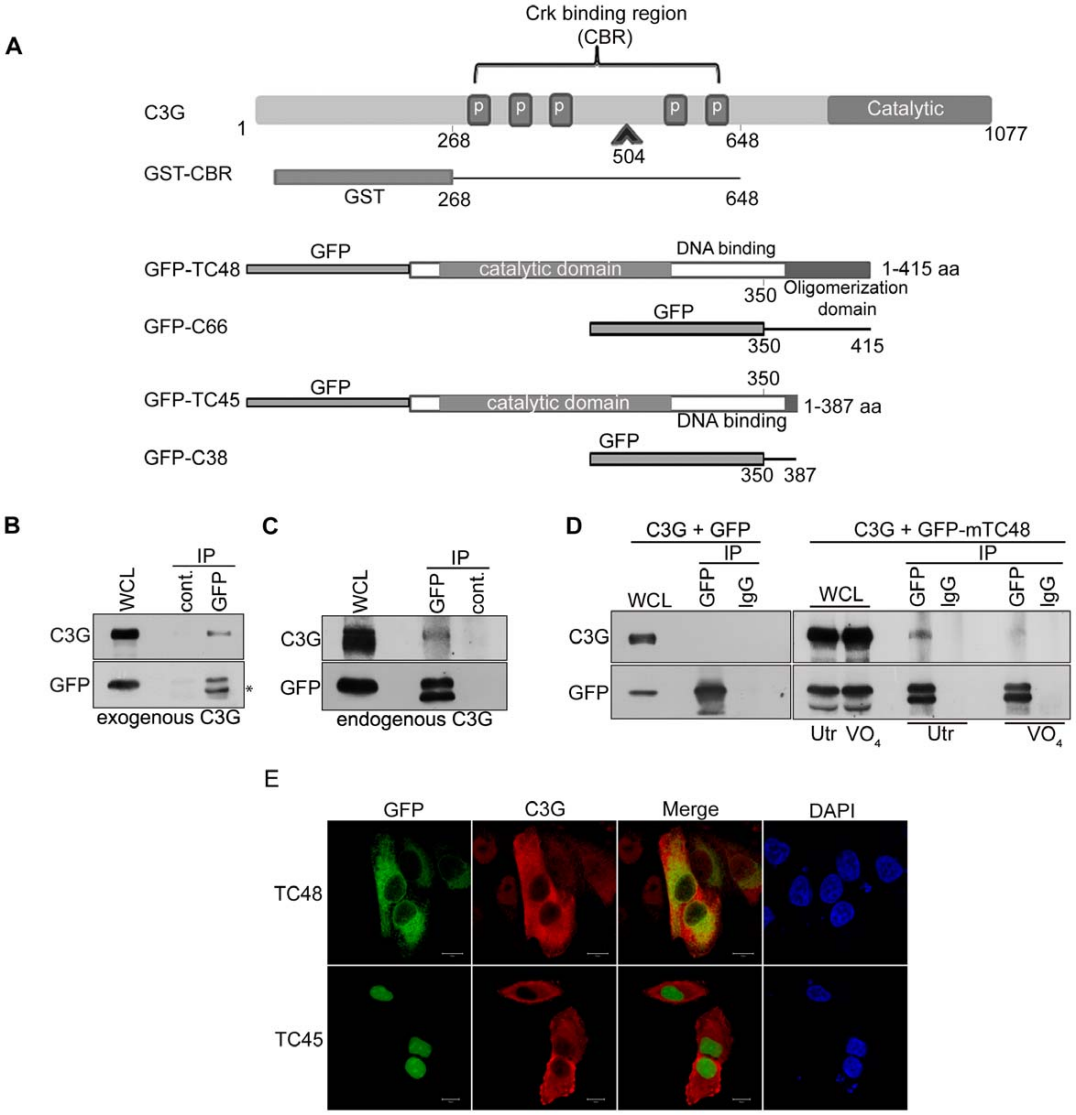
C3G interacts with TC-PTP: (A) Schematic showing domain organization of C3G and the two splice variants of TC-PTP, TC48 and TC45 as GFP fusion proteins. Fusion proteins of GFP with C-terminal sequence of TC48 and TC45 are also shown. Recombinant GST fusion protein of the Crk binding region of C3G is also shown (GST-CBR). (B) C3G co-precipitates with mTC48 from cell lysates. HEK-293 cells transfected with C3G and GFP-mTC48 were subjected to immunoprecipitation using control mouse IgG or GFP antibodies. The immunoprecipitates were analyzed for western blotting with C3G and GFP antibodies. Whole cell lysates (WCL) used for immunoprecipitation are also included to show expression of the protein. * indicates partially degraded mTC48 in lysates during immunoprecipitation. (C) Interaction of mTC48 with cellular C3G. Experiment was carried out as in (B) using lysates of cells transfected with mTC48 alone. (D) Interaction of mTC48 with C3G is abrogated by vanadate. GFP-mTC48 was immunoprecipitated from lysates of HEK293 cells expressing C3G and GFP-mTC48, left untreated (Utr) or treated with 50µM vanadate (VO_4_) for 4 hrs. Cells were subjected to 10 min treatment with PV to stimulate C3G phosphorylation prior to lysis. Along with lysates (WCL), the immunoprecipitates were subjected to Western blotting with the indicated antibodies. Immunoprecipitation was also carried out from cells expressing GFP and C3G as a control. (E) Sub-cellular localization of C3G and TC-PTP. IMR-32 cells transfected with C3G and either GFP-TC48 or GFP-TC45 were stained for C3G and analyzed by confocal microscopy. A section through the centre of the nucleus is shown. Bar, 10 µm.

As a first step towards exploring the functional regulation of C3G by TC-PTP, possible interaction between C3G and TC48 was tested by examining the ability of C3G to co-precipitate with a catalytically inactive substrate trap mutant, mTC48 (D182A). Mutant enzymes have been used to study interaction between tyrosine phosphatases and their substrates [37]. It was seen that exogenously expressed (Fig. 1B) as well as cellular C3G (Fig. 1C) co-precipitated with the mutant enzyme, indicating that C3G and TC48 interact physically *in vivo*. We have earlier shown that cellular C3G is phosphorylated on Y504 upon treatment of cells with pervanadate (PV) [18]. In cells stimulated with PV, interaction between C3G and mTC48 was competed out by sodium orthovanadate, a phosphotyrosine mimetic, suggesting direct binding (Fig. 1D). No interaction is seen between overexpressed C3G and GFP used as a control.

TC48 has earlier been shown to localize to the Golgi and ER [28], [29]. In IMR-32 cells too, it was observed that TC48 shows predominant localization in the Golgi region of the cell as evidenced by co-staining with Golgi marker, Giantin and ERGIC marker, ERGIC53 (Fig. S2). We also examined the possible colocalization of C3G and TC48 in human neuroblastoma cells. Cells co-expressing C3G with TC48, showed minimal colocalization in the cytoplasmic compartment in confocal sections through the centre of the nucleus. While TC48 showed juxta nuclear prominence, C3G was distributed throughout the cytoplasm with weaker presence in the area of the Golgi (Fig. 1E). This pattern suggested that C3G does not localize at the Golgi and is mostly cytosolic. C3G and the TC45 variant showed mutually exclusive localization with the later present only in the nucleus.

**Figure 2:**
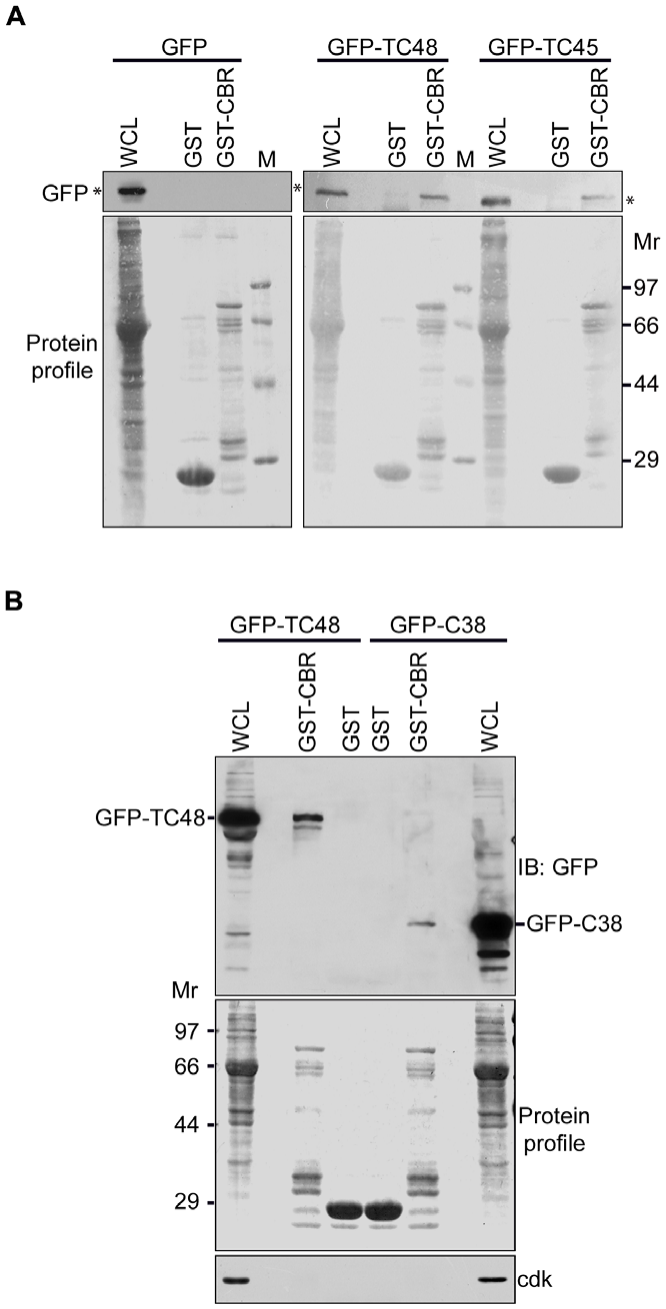
C-terminal residues of TC-PTP are responsible for direct interaction with CBR domain of C3G. (A) *In vitro* interaction between C3G and TC-PTP. GST and GST-CBR coupled to glutathione agarose beads were incubated with lysates of HEK-293 cells expressing GFP, GFP-TC45 or GFP-TC48. The WCL and washed beads were subjected to western blotting for GFP. The recombinant protein levels are shown in the Ponceau stained blot. M, protein molecular weight markers. * indicates position of GFP and the GFP fusion proteins. (B) C3G Crk binding region interacts with C-terminal regulatory sequences of TC-PTP. TC48 and a deletion construct of TC45, GFP-C38 (schematic shown in Fig. 1A) were used in *in vitro* interaction assay as described in (A).

Ability of C3G and TC48 to interact directly was examined in an *in vitro* pull down assay using a fusion protein of GST coupled to the Crk binding region of C3G encompassing 268-648 residues of C3G (GST-CBR), shown in Fig. 1A. Lysates of HEK293 cells expressing GFP, GFP-TC48 or GFP-TC45 were incubated with GST or GST-CBR coupled to glutathione-agarose beads. As shown in Fig. 2A, GFP-TC48 and GFP-TC45 interacted specifically with GST-CBR but not with GST alone. GFP did not interact with GST or the recombinant fusion protein. TC48 and TC45 differ in their C-termini, with TC48 showing a stretch of hydrophobic sequences through which it is known to oligomerize and interact specifically with other proteins [27], [29], [40]. Using a deletion construct of TC48, GFP-C66 in *in vitro* pull down assays, we observed that C-terminal residues of TC48 (350-416) are sufficient for interaction with C3G (Fig. S1). Since TC45 also showed interaction in these assays it could be inferred that the hydrophobic unique residues in TC48 are not essential for the interaction. These results implied that residues between 350 and 381 at the C-terminus of TC-PTP which are present in both isoforms may be responsible for interaction with CBR domain of C3G. This was examined by testing for interaction between a GFP fusion protein containing C-terminal 38 residues of TC45 (350-387) with GST-CBR. Fig. 2B shows that C-terminal 38 residues in TC45 are sufficient for specific interaction with C3G-CBR. The absence of non-specific interaction was seen by the lack of interaction of GST with C38 and also lack of interaction of GST-CBR with a cellular protein, Cdk2. From the multiple *in vitro* interaction assays performed, it was seen that the C-terminal deletion constructs showed weaker affinity for GST-CBR compared to the full length PTP isoforms.

**Figure 3:**
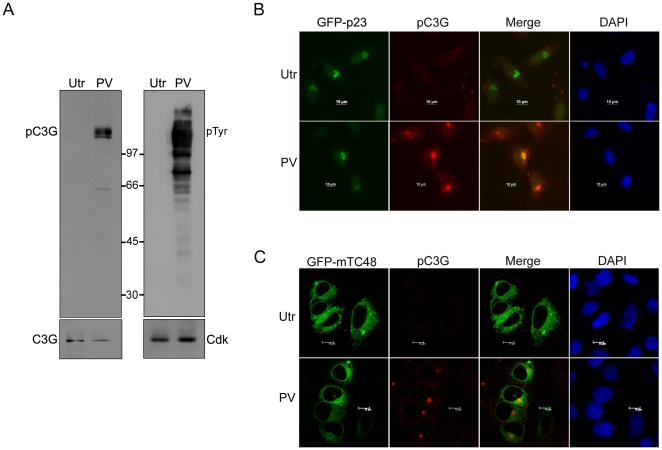
Pervanadate (PV) treatment induces Y504 phosphorylation of C3G and pC3G localizes to the Golgi. (A) IMR-32 cells were subjected to PV treatment and lysates used in Western blotting for pC3G, C3G, pTyr and Cdk2 (as loading control). (B) IMR-32 cells growing on coverslips were transfected with GFP-p23, subjected to PV treatment and stained for pC3G. Panels show images of cells captured using a fluorescence microscope fitted with an apotome. Bar, 10 µm. (C) pC3G co-localizes with TC48 at the Golgi. IMR-32 cells expressing GFP- mTC48 were subjected to PV treatment and stained for pC3G. Panels show a single section in the Z-axis captured using a confocal microscope. Utr, untreated, PV, pervanadate treated. Bar, 10 µm.

Endogenous C3G is phosphorylated on Y504 in response to forskolin or NGF treatment of IMR-32 cells and pC3G shows Golgi localization [12]. This phosphorylation was shown to be mediated by SFKs. IMR-32 cells subjected to PV treatment which activates SFKs, also induced phosphorylation of endogenous C3G specifically on Y504, detected using the phospho-specific antibody pY504-C3G (Fig. 3A). This antibody does not interact with the large number of other cellular proteins phosphorylated on tyrosine in response to PV treatment, detected using pTyr antibody. The specificity of this antibody to detect C3G phosphorylated on Y504 but not other tyrosine residues was also shown by immunoprecipitating C3G and Y504F mutant from cells co-expressing c-Src tyrosine kinase and immunoblotting with pC3G and pTyr antibodies. Fig. S3A shows that the pC3G antibody detects only WT C3G phosphorylated by Src, but not the Y504F mutant. This mutant showed reactivity with pTyr antibodies indicating that tyrosine residues of C3G other than Y504 are phosphorylated by Src. The specificity of the pC3G antibody to detect C3G phosphorylated on Y504 in indirect immunofluorescence is shown in Fig. S3B. PV activated cellular kinases to phosphorylate both endogenous as well as overexpressed C3G (detected as cytoplasmic staining corresponding with C3G expression) whereas overexpressed Y504F mutant expressing cells showed no enhanced pC3G staining.

Endogenous pC3G localization was examined by indirect immunofluorescence in cells transfected with GFP- p23, a protein that localizes to the Golgi [29]. PV induced pC3G co-localized with GFP- p23 at the Golgi (Fig. 3B). The colocalization of pC3G with TC48 was tested in cells transfected with a catalytically inactive mutant TC48 (mTC48) upon treatment with PV. As seen in Fig. 3C, pC3G colocalizes with mTC48 in the core of the Golgi. PV induced phosphorylation of C3G was reversible and subject to the action of cellular tyrosine phosphatases. Upon removal of PV and incubation with cDMEM for 4 hours, cells showed loss of pC3G staining (Fig. S4). pC3G staining was more prominent when PV treated cells were incubated with cDMEM containing 50 µM VO_4_ indicating that cellular tyrosine phosphatases could dephosphorylate C3G.

### TC48 expressing cells do not show pC3G induced by PV or SFKs

**Figure 4:**
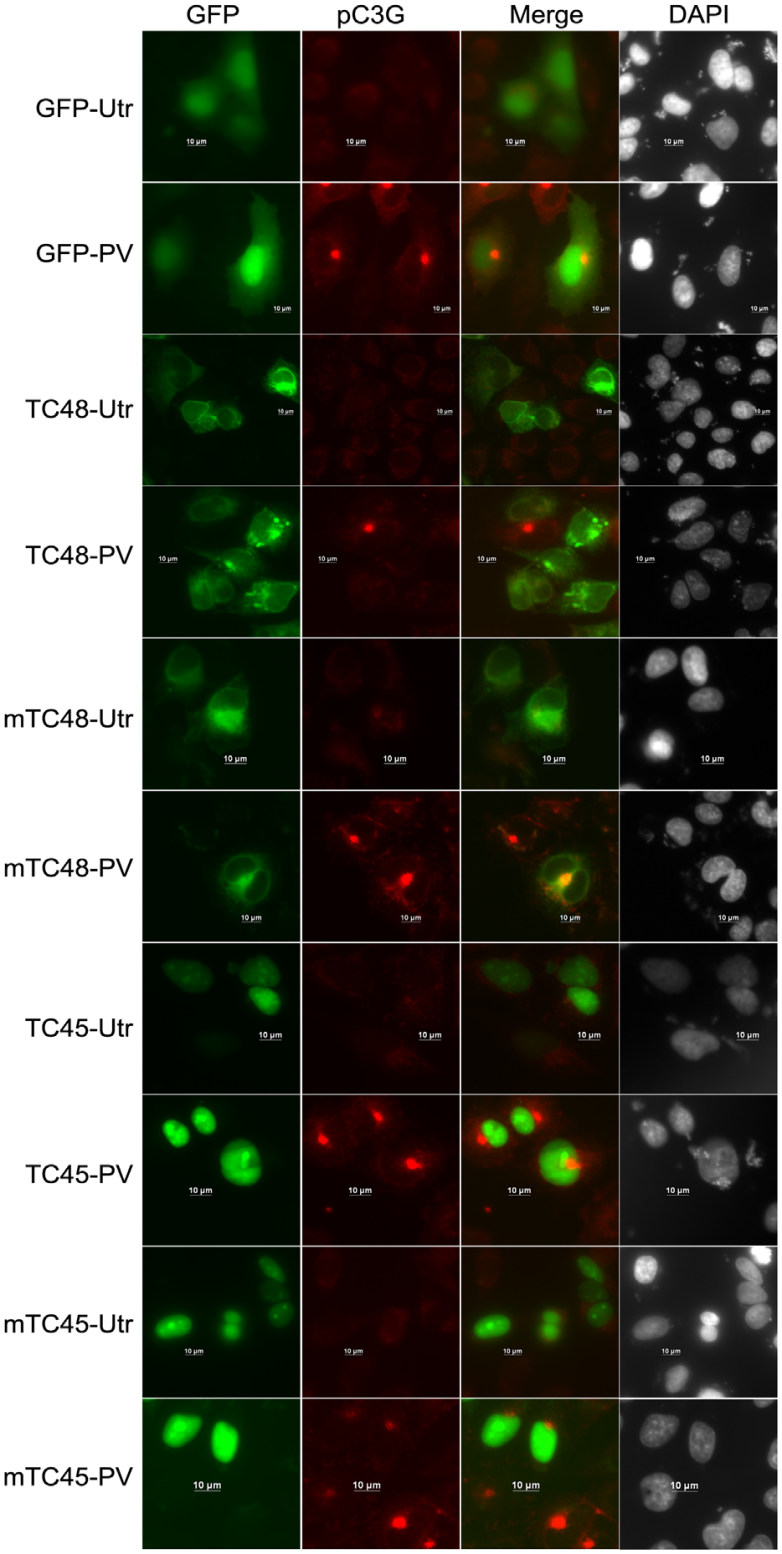
TC48 expression inhibits PV induced C3G phosphorylation. IMR-32 cells expressing GFP, GFP-TC48, GFP-mTC48, GFP-TC45 or GFP-mTC45 were subjected to PV treatment and stained for pC3G. Utr, Untreated. Bar, 10 µm.

Co-immunoprecipitation experiments which showed interaction between C3G and the substrate trap mutant phosphatase and the sensitivity of this interaction to vanadate, suggested that C3G may be a substrate of TC-PTP. This was examined by observing the presence of pC3G in IMR-32 cells expressing either GFP, or GFP tagged TC45, mTC45, TC48 & mTC48 after being subjected to PV treatment. As shown in Fig. 4, cells expressing GFP, TC45, mTC45 or mTC48 showed no affect on pC3G staining, whereas TC48 expressing cells lacked the presence of pC3G in the Golgi. Cells that were not treated with PV showed total absence of any pC3G staining. These results indicated that Golgi localized TC48 may be dephosphorylating Y504 phosphorylated C3G induced by PV treatment, but the nuclear TC45, does not act on C3G. Expression of HA-TC45, that can exit the nucleus in response to certain stimuli, also did not affect PV induced cytoplasmic phospho-C3G (Fig. S5A). Under similar conditions HA-TC48 caused dephosphorylation of C3G.

To rule out a possibility for cell type specific effects with respect to targets of TC-PTP, the ability of TC48 to dephosphorylate C3G was also examined in Cos-1 cells. PV treatment resulted in phosphorylation of C3G in cells expressing TC45 or mTC48, but not in cells expressing wild-type TC48 (Fig. S5B). No difference was seen in overall pTyr staining induced by PV in cells expressing TC45 or TC48 compared to non-expressing cells (Fig. S5C). These results indicated the specificity of C3G as a substrate for TC48 and that TC48 does not act on cellular pTyr in general due to overexpression.

**Figure 5:**
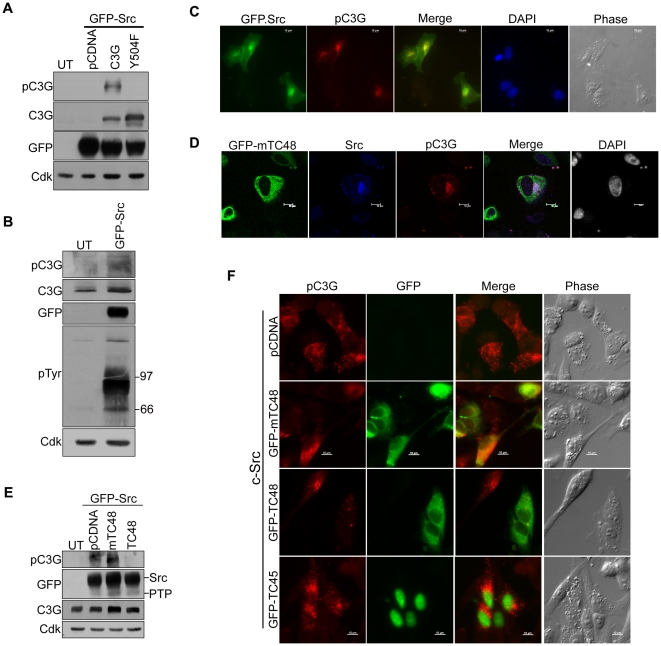
TC48 inhibits c-Src induced C3G phosphorylation. (A) Lysates of HEK293 cells expressing GFP-Src along with either pCDNA, C3G or Y504F mutant were subjected to western blotting with the indicated antibodies. (B) IMR-32 cells transfected with GFP-c-Src were subjected to western blotting to detect endogenous C3G phosphorylated on Y504. Blots were exposed longer compared to those in 4A, where endogenous pC3G is not detected. (C) GFP-c-Src expressing IMR-32 cells were stained for pC3G to visualize localization pattern. (D) IMR-32 cells transfected with GFP-mTC48 and c-Src were stained for indirect immunofluorescence using Src and pC3G antibodies. (E) GFP-c-Src was co-expressed with either pCDNA, GFP-mTC48 or GFP-TC48 in IMR-32 cells and lysates subjected to western blotting as indicated. (F) *In situ* expression of pC3G in IMR-32 cells expressing c-Src along with pCDNA, GFP-mTC48, GFP-TC48 or GFP-TC45. Bar, 10 µm.

Neurotrophin signaling is mediated through Src and we have earlier shown that SFKs can phosphorylate C3G at Y504 which then predominantly localizes to the Golgi [12], [18]. c-Src expression was therefore used to study the dynamics of endogenous C3G phosphorylation. Y504 phosphorylated C3G was seen only in lysates of cells co-expressing WT-C3G with Src and not in Y504F mutant expressing cells (Fig. 5A). GFP-c-Src expression in IMR-32 cells, resulted in phosphorylation of endogenous C3G detected in Western blots by giving longer exposures (Fig. 5B). GFP-c-Src showed prominent Golgi localization and Src expressing cells show the presence of pC3G in the Golgi (Fig. 5C). In c-Src and mTC48 expressing cells, pC3G colocalizes with these two proteins in the juxtanuclear region (Fig. 5D). The ability of TC-PTP to dephosphorylate C3G phosphorylated by c-Src was tested by co-expressing c-Src with either pCDNA or the TC-PTP constructs and observing the presence of pC3G in indirect immunofluorescence and western blotting. As shown in Fig. 5E, lysates from cells expressing c-Src with pCDNA showed the presence of pC3G, but lysates from cells co-expressing TC48 did not show the presence of any pC3G. Cells expressing mTC48 showed higher levels of pC3G relative to control lysates indicating that mTC48 may be inhibiting the activity of endogenous TC48 by functioning as a dominant negative. Expression of GFP-Src, GFP-TC48 and GFP-mTC48 was confirmed by probing the blot with GFP (Fig. 5E). Examination of cells expressing c-Src also showed the absence of pC3G in wild type TC48 co-expressing cells whereas TC45 expression did not affect pC3G staining (Fig. 5F). Dephosphorylation of pC3G by TC48 appeared to be specific since pTyr staining induced by c-Src on other cellular proteins was not affected by expression of either TC48 or TC45 (Fig. S6).

**Figure 6:**
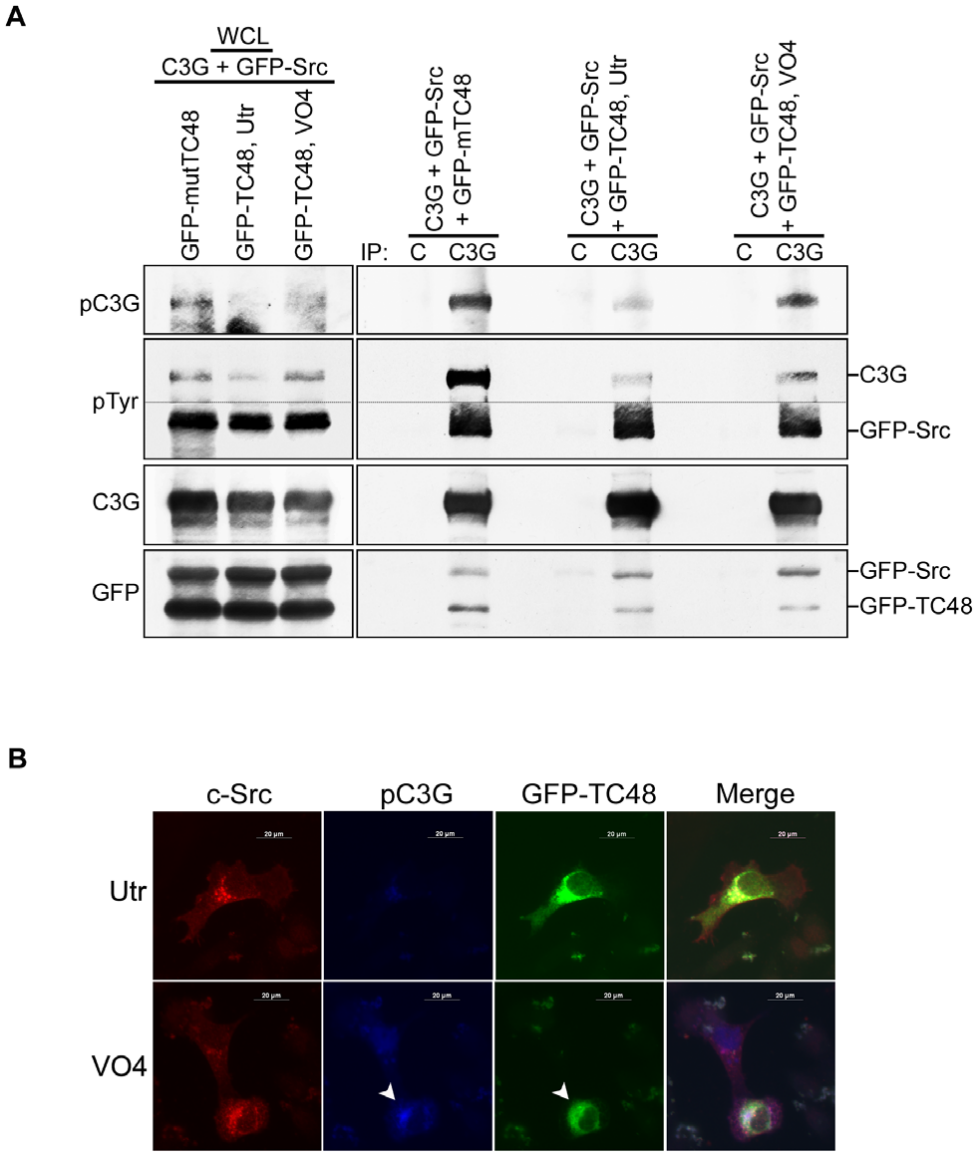
TC48 mediated C3G dephosphorylation is abrogated by vanadate. (A) C3G was immunoprecipitated from lysates of HEK293 cells expressing C3G and GFP-Src with either TC48 or mTC48. Immunoprecipitation in TC48 expressing cells was carried out in the presence (VO4) or absence (Utr) of sodium vanadate treatment. Along with lysates (WCL), the immunoprecipitates were subjected to Western blotting with the indicated antibodies. A dotted line has been added in the pTyr blot to indicate that probing for the blots corresponding to C3G and Src were carried out separately. C, control IgG. (B) IMR-32 cells expressing c-Src and GFP-TC48 were left untreated (Utr) or treated with vanadate (VO4) prior to fixation and staining for c-Src and pC3G. In the presence of vanadate, TC48 expressing cell (arrow) shows pC3G.

C3G dephosphorylation due to action of TC48, was confirmed by treating TC48 expressing cells with VO_4_ for 4 hrs prior to lysate preparation or fixation. The phosphorylation of C3G was examined in C3G immunoprecipitates from cells expressing C3G along with GFP-Src, mutant or WT TC48. Src as well as TC48 were seen in C3G immunoprecipitates suggesting that C3G could form a trimolecular complex containing both the kinase and the phosphatase in cells (Fig. 6A). Co-expression of WT TC48 decreased Y504 phoshorylation of C3G significantly compared to that seen in mutant TC48 expressing cells.

VO_4_ treatment inhibited the ability of TC48 to dephosphorylate C3G. TC48 expression did not affect p-Tyr levels of Src. It was observed that mTC48 interacts better with C3G compared to the WT enzyme. No significant difference was seen in interaction of WT TC48 with C3G in the presence of VO_4_. pC3G was detected by indirect immunoflourescence in c-Src expressing cells irrespective of the presence or absence of TC48 expression in vanadate treated cells (Fig. 6B).

### c-Src induced pC3G is dephosphorylated by endogenous TC48

**Figure 7:**
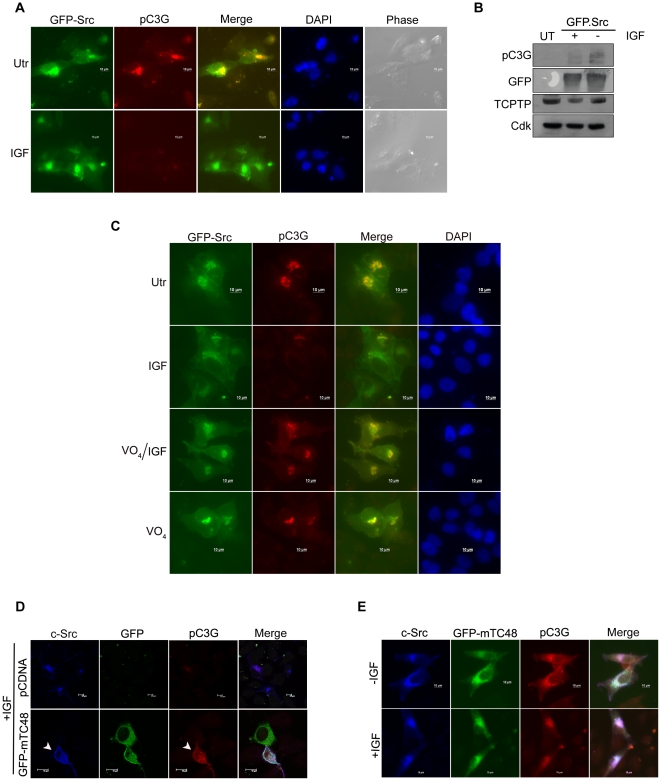
IGF mediated dephosphorylation of C3G is dependent on endogenous TC48. (A) GFP-c-Src expressing IMR-32 cells were treated with IGF prior to fixation and staining for pC3G. Utr, untreated. (B) Lysates of cells treated as in (A) were subjected to western blotting using the indicated antibodies. (C) Pre-treatment with VO_4_ abrogates IGF induced dephosphorylation of C3G. GFP-c-Src expressing cells were subjected to IGF treatment with or without VO_4_ pre-treatment for 30 min and stained for pC3G. Cells treated with VO_4_ alone are also shown. Bar, 10 µm. (D) Expression of GFP-mTC48 along with c-Src, inhibits IGF induced dephosphorylation of C3G. IMR-32 cells were co-transfected with c-Src, and either pCDNA or GFP-mTC48, subjected to IGF treatment and stained for Src and pC3G. Arrow indicates a cell co-expressing mTC48 along with c-Src showing pC3G. (E) mTC48 expressing cells show Src induced pC3G irrespective of their exposure to IGF.

Treatment of cells with insulin-like growth factor (IGF) is known to induce the activity of cellular tyrosine phosphatases to modulate signaling pathways [41], [42]. The ability of endogenous tyrosine phosphatases to dephosphorylate pC3G was examined by subjecting IMR-32 cells transfected with c-Src to IGF treatment. As shown in Fig. 7A & B, IGF treatment resulted in significant reduction in pC3G levels. Pretreatment of cells with VO_4_ for 30 min prior to IGF treatment restored pC3G staining, indicating that IGF induced repression of pC3G was due to the activity of cellular tyrosine phosphatases (Fig. 7C). Examination of total cellular pTyr levels showed no significant difference upon IGF treatment in a majority of Src induced tyrosine phosphorylated proteins indicating that pC3G may be selectively targeted by cellular PTPases in response to IGF (Fig. S7).

To examine the contribution of endogenous TC48, we tested the presence of pC3G in c-Src expressing cells co-transfected with mTC48. Catalytically inactive TC-PTP constructs have been earlier shown to act as dominant negatives to inhibit the function of endogenous TC48 [38], [40]. We therefore examined pC3G levels in mTC48 expressing cells upon IGF treatment. Cells co-expressing c-Src with mTC48 showed pC3G staining upon IGF treatment unlike cells expressing control vector (Fig. 7D). pC3G staining was seen in mTC48 expressing cells in the presence or absence of IGF (Fig. 7E). These results suggested that endogenous TC-PTP could be stimulated to dephosphorylate endogenous C3G upon IGF treatment.

### TC48 inhibits forskolin induced C3G phosphorylation and neurite growth

**Figure 8:**
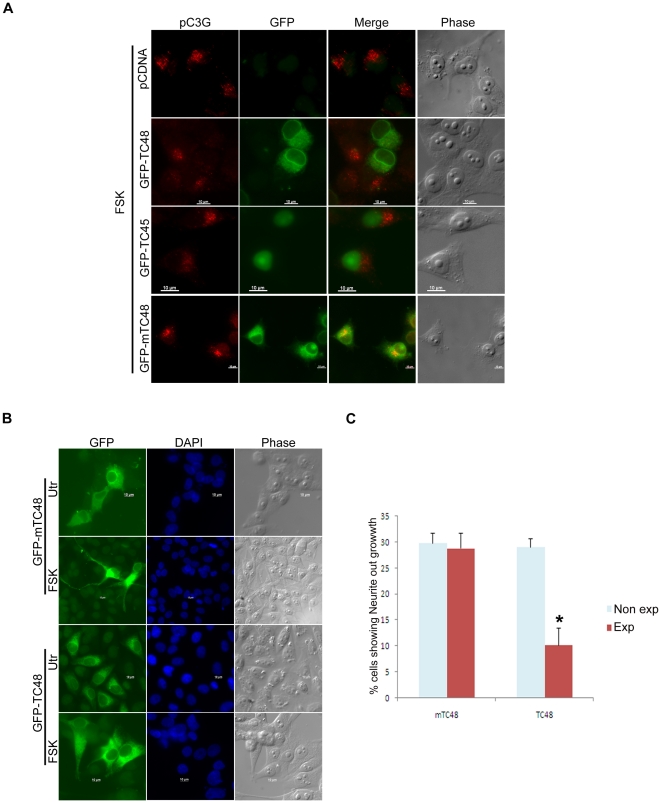
TC48 expression inhibits forskolin induced phosphorylation of C3G at the Golgi and neurite growth. (A) IMR-32 cells transfected with pCDNA, GFP-TC45, GFP-TC48 or GFP-mTC48 were treated with forskolin for 2 hrs, fixed and stained for pC3G. Bar, 10 µm. (B) IMR-32 cells transfected with GFP-TC48 or GFP-mTC48 were left to grow in normal medium (Utr) or induced to differentiate by treatment with forskolin for 72 hrs (FSK). Cells were fixed and observed for neurite growth in GFP positive cells using a fluorescence microscope. (C) Quantitation of neurite growth in GFP positive and negative cells after transfection with GFP-TC48 and GFP-mTC48 upon forskolin treatment. *p<0.01.

We have earlier shown that C3G is phosphorylated at the Golgi dependent on endogenous SFK activation upon treatment of IMR-32 cells with Fsk or NGF [12]. We therefore examined the ability of TC-PTP to dephosphorylate C3G phosphorylated in response to Fsk treatment. As shown in Fig. 8A, TC48 expressing cells failed to show any pC3G in the Golgi unlike cells expressing control vector, TC45 or mTC48. Fsk acts as a neurotrophin to induce differentiation of IMR-32 cells and SFKs are known to participate in the signaling responses [43]. To determine whether TC48 overexpression, (because of its ability to repress C3G phosphorylation), can affect neurite outgrowth induced by Fsk, IMR-32 cells transfected with mTC48 or wild type TC48 were subjected to Fsk treatment for 72 hrs to enable differentiation. GFP expressing and non-expressing cells from the various fields were examined for neurite growth. No difference was seen in cell morphology between expressing and non-expressing cells not treated with Fsk. In Fsk treated cells, mTC48 expression did not affect the ability of Fsk to induce neurites, whereas TC48 expressing cells were significantly compromised in their ability to grow neurites (Fig. 8B,C). These results indicate that TC48 overexpression can disrupt signaling induced by Fsk and disable neuronal differentiation.

## Discussion

Results from this study provide evidence for the identification of C3G as an interacting partner and substrate of TC48. The ability of TC-PTP to dephosphorylate C3G is dependent on its sub-cellular localization since expression of the nuclear isoform, TC45 did not dephosphorylate C3G. Distinct sub-cellular localization to defined cellular compartments often defines the role of PTPases in specific biological functions and this is the first report of a Golgi localized substrate for TC48. Evidence for C3G being a substrate of TC48 has been provided based on the following findings: (1) Better interaction between C3G and catalytically inactive TC48 which functions as a substrate trapping mutant, compared to WT TC48 within mammalian cells. (2) Inhibition of interaction between C3G and mTC48 by the phosphotyrosine mimetic, sodium vanadate. (3) TC48 expression causes dephosphorylation of endogenous C3G, but does not affect overall tyrosine phosphorylation in cells. (4) Inhibition of cellular TC48 function using a dominant negative mutant disables IGF mediated dephosphorylation of C3G. (5) Vanadate treatment inhibits TC48 mediated dephosphorylation of C3G.

C3G and TC48 appear to occupy distinct compartments, with C3G showing a more diffused staining with higher levels in the sub-cortical region and TC48 localizing in the nuclear membrane, Golgi and ER. Nevertheless, C3G and TC48 complexes form in the cellular context. Though C3G is distributed throughout the cytoplasm, PV or Fsk induced pC3G staining is seen in the core of the Golgi, indicating that either Golgi localized tyrosine kinases mediate C3G phosphorylation *in vivo* or that phophorylated C3G is targeted to the Golgi. Effectors of C3G function like Rap1 are known to localize to the Golgi and have also been shown to be specifically activated in distinct sub-cellular compartments [44]. Regulation of C3G activity at the Golgi could therefore be mediated by dynamic shuttling of TC48 between the Golgi and ER compartments aided through interaction with cargo receptors p23 and p25 [29]. This may be a mechanism of regulating spatio-temporal changes required for Golgi mediated functions during neuronal differentiation [45]. c-Src localizes to the Golgi and is also activated by PV and Fsk treatment [12], [18]. Src induced NOG is mediated by recruitment of Crk, a molecule that is constitutively associated with C3G in cells [46]. We have therefore used c-Src expression for studying phosphorylation dynamics of endogenous C3G in the presence or absence of TC48. The lack of c-Src induced pC3G in TC48 expressing cells may also be due to inactivation of c-Src by TC-PTP [47]. Our observation of pTyr staining that matched c-Src expression in TC48 or TC45 expressing cells indicated that TC48 may be specifically dephosphorylating C3G and not altering c-Src kinase activity in IMR-32 cells. Overexpression of PTPases is likely to cause dephosphorylation of targets that may not be relevant as physiological substrates. Our results showing that IGF treatment induces dephosphorylation of C3G dependent on endogenous TC48 provided evidence for C3G being a physiological substrate of TC48.

Many TC-PTP substrates have been identified using substrate-trap approaches, wherein interaction between the phosphatase and its target is mediated through the catalytic domain and is dependent on substrate phosphorylation. Proteins like p97, p23, p25 and integrin α1, known to associate with non-catalytic sequences of TC-PTP, function in regulating its activity and localization [29], [48], [49]. Using *in vitro* binding assays, we have identified that the C-terminal sequences of TC-PTP (350-381 amino acids) are sufficient for direct interaction with Crk binding region of C3G. This region of C3G is known to interact with SH3 domain containing molecules. Our study shows that it may also be capable of other types of previously undefined interactions. That stable interaction between full length TC-PTP and C3G was partially dependent on the catalytic domain was indicated by results showing that: 1) wild type TC48 differed in interaction with C3G compared to its catalytically inactive mutant, and 2) reduced interaction between mTC48 and phosphorylated C3G in the presence of vanadate. It therefore appears that the catalytic domain of TC-PTP may aid in the stability of interaction with C3G, primarily mediated through its non-catalytic sequences. This was evident from the fact that full length TC-PTP isoforms interacted better with C3G-CBR in *in-vitro* assays compared with the deletion constructs with only C-terminal sequences.

Neurotransmitter induced neurite growth involves complex and coordinated participation of a number of signaling proteins [50], [51]. A large number of cellular proteins are phosphorylated on tyrosine during neuronal differentiation. PTPases have been shown to be involved in neurite growth and differentiation [52], [53]. PTP sigma dephosphorylates neurotrophin receptors and suppresses NOG [53]. C3G has been shown to be essential for neuronal differentiation and knock-out animals show enlarged brain cortical regions [25]. C3G is phosphorylated in response to forskolin and NGF and TC-PTP expression inhibited forskolin induced differentiation of neuroblastoma cells dependent on its catalytic activity. TC-PTP may be acting through dephosphorylation of substrates required during neurite growth, one of them being C3G. Expression of RPTP gamma was shown to reduce levels of a 140 kDa protein and inhibit NOG induced by NGF in PC12 cells [54]. Since C3G is phosphorylated on tyrosine in response to NGF, it is possible that the 140 kDa protein may be C3G. Overexpressed TC-PTP may be regulating C3G effector functions, thereby inhibiting neuronal differentiation. Earlier work showing that levels of TC-PTP are low in adult brain also suggest a negative role for TC-PTP in maintaining differentiation [55]. A role for TC-PTP has been implicated in IGF induced phototransduction in retinal rod cells [56]. Thus far, no defects in development and maturation of the nervous system have been described in TC-PTP knock-out mice. Both TC-PTP and C3G show ubiquitous expression and are known to be involved in diverse signaling pathways. C3G may be one of the substrates regulated by TC-PTP to maintain cellular homeostasis.

These results describe the identification of the first intracellular tyrosine phosphatase in the regulation of C3G. Interaction with, and dephosphorylation by TC48 represents a novel mechanism of C3G regulation that is physiologically significant in the context of neurite growth in human neuroblastoma cells.

## Supporting Information

Figure S1 C3G Crk binding region interacts with C-terminal regulatory sequences of TC-PTP. TC45 and a deletion construct of TC48, GFP-C66 (schematic shown in Fig. 1A) were used in *in vitro* interaction assay. GST and GST-CBR coupled to glutathione agarose beads were incubated with lysates of HEK-293 cells expressing GFP-TC45 or GFP-C66. The WCL and washed beads were subjected to western blotting for GFP. The recombinant protein levels are shown in the Ponceau stained blot. M, protein molecular weight markers. * indicates position of GFP-TC45 and the GFP-C66 fusion proteins.
(TIF)

Figure S2 TC48 localizes predominantly to the ER & Golgi in IMR-32 cells. IMR-32 cells expressing GFP-TC48 were stained for Giantin (Golgi marker) (A) or ERGIC-53 (ER-Golgi intermediate compartment marker) (B). Panels show images of a single section captured using 63X objective of Leica Confocal Microscope (A) or using 40X Objective of Carl Zeiss, Axioimager Z1 upright microscope. Bar, 10 µm.
(TIF)

Figure S3 pY504-C3G antibody specifically recognizes C3G phosphorylated on Y504. (A) Lysate of HEK293 cells expressing GFP-Src with either WT C3G or Y504F mutant were subjected to immunoprecipitation using control IgG or C3G antibody. The immunoprecipitates were subjected to Western blotting along with cell lysates (WCL) using the antibodies against pY504 C3G, C3G and pTyr. The pC3G antibody recognizes WT-C3G from cells co-expressing GFP-Src. No reaction is seen with Y504F mutant, which is recognized by pTyr antibodies indicating that this mutant is phosphorylated on tyrosine residues other than Y504. A dotted line has been added in the pTyr blot to indicate that probing of the blots corresponding to C3G and Src were carried out seperately. Level of C3G is shown for comparison. (B) Specificity of pC3G antibody to detect pY504 C3G *in situ* by indirect immunofluorescence. IMR-32 cells were transfected with C3G or Y504F mutant and left untreated (Utr) or subjected to PV treatment (50 µM, 10 mins) and stained for C3G and pC3G. Endogenous pC3G is seen in the Golgi and membrane of all PV treated cells. WT C3G expressing cells show enhanced pC3G staining in pervanadate (PV) treated cells whereas Y504F mutant expressing cells do not. C3G and Y504F expressing cells are indicated by arrows. No pC3G signal is seen in untreated cells. Y504F mutant expressing cells show slightly weaker staining for endogenous pC3G by virtue of its ability to function as a dominant negative to inhibit phosphorylation of endogenous C3G. Bar 10 µm.
(TIF)

Figure S4 pC3G is dephosphorylated by cellular tyrosine phosphatases. IMR-32 cells were treated with PV (10 min, 50 µM) and left in normal medium (cDMEM) or medium containing 50 µM sodium vanadate for 4 hrs. Cells were fixed and stained for pC3G. Cells left in cDMEM show loss of pC3G whereas enhanced levels of pC3G were seen upon exposure to medium containing VO4. Bar, 10 µm. Utr: untreated cells.
(TIF)

Figure S5 TC48 inhibits PV induced pC3G in IMR32 and Cos-1 cells: (A) IMR32 cells expressing HA-TC45 or HA-TC48 were left untreated (Utr) or subjected to PV treatment and stained for pC3G. TC45 expression has no effect on pC3G while TC48 expressing cells showed decreased pC3G staining. Bar, 10 µm. (B,C) Cos-1 cells expressing GFP-TC45 or GFP-TC48 were left untreated (Utr) or subjected to PV treatment and stained for pC3G (B) and p-Tyr (C). While TC45 expression has no effect on either pC3G or pTyr staining in cells, TC48 expressing cells showed significantly less pC3G, but no difference in pTyr staining.
(TIF)

Figure S6 TC-PTP expression does not affect c-Src induced cellular pTyr. IMR-32 cells were transfected with c-Src along with either GFP, GFP-TC48, GFP-mTC48, or GFP-TC45. Fixed cells were stained to visualize expression of Src and pTyr. Images show fields captured using a fluorescence microscope. Bar, 10 µm.
(TIF)

Figure S7 IGF treatment does not affect Src induced pTyr on most cellular proteins. IMR-32 cells expressing GFP-Src were either left untreated or subject to 100 nM IGF treatment prior to preparation of whole cell lysates. Cell lysates were subject to Western blotting to detect total cellular pTyr levels. GFP blotting was performed to indicate expression of GFP-Src and CDK2 as loading control. UT, untransfected. pTyr band corresponding to C3G is indicated.
(TIF)
