# Supplementary figures and images for: TC-PTP Dephosphorylates the Guanine Nucleotide Exchange Factor C3G (RapGEF1) and Negatively Regulates Differentiation of Human Neuroblastoma Cells
Source: PLoS One. 2011 Aug 18;6(8):e23681. doi: 10.1371/journal.pone.0023681 (PMC3158094; doi:10.1371/journal.pone.0023681)

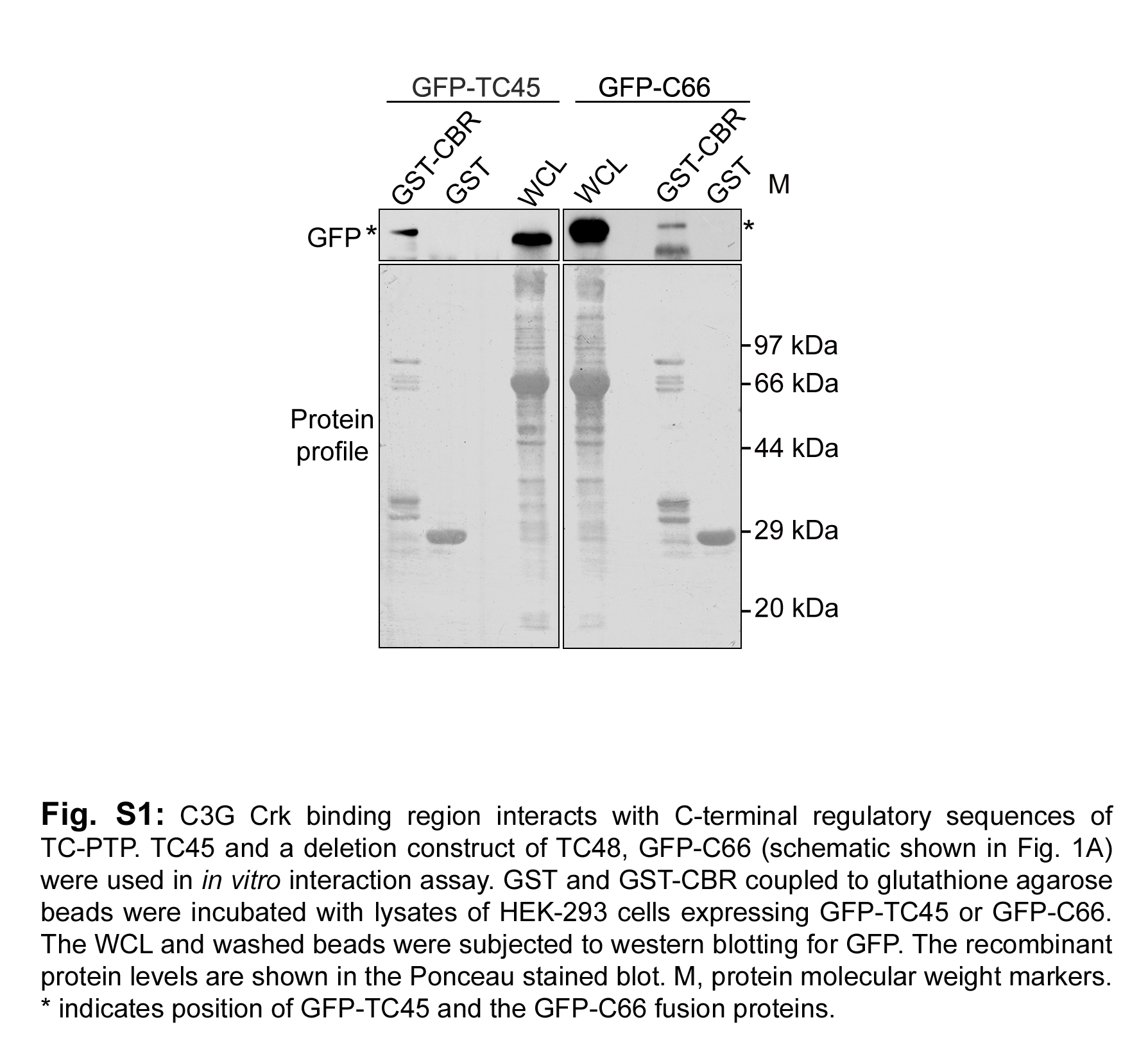

Supplement: Figure S1 — C3G Crk binding region interacts with C-terminal regulatory sequences of TC-PTP. TC45 and a deletion construct of TC48, GFP-C66 (schematic shown in Fig. 1A) were used in in vitro interaction assay. GST and GST-CBR coupled to glutathione agarose beads were incubated with lysates of HEK-293 cells expressing GFP-TC45 or GFP-C66. The WCL and washed beads were subjected to western blotting for GFP. The recombinant protein levels are shown in the Ponceau stained blot. M, protein molecular weight markers. * indicates position of GFP-TC45 and the GFP-C66 fusion proteins. (TIF) [file pone.0023681.s001.tif]

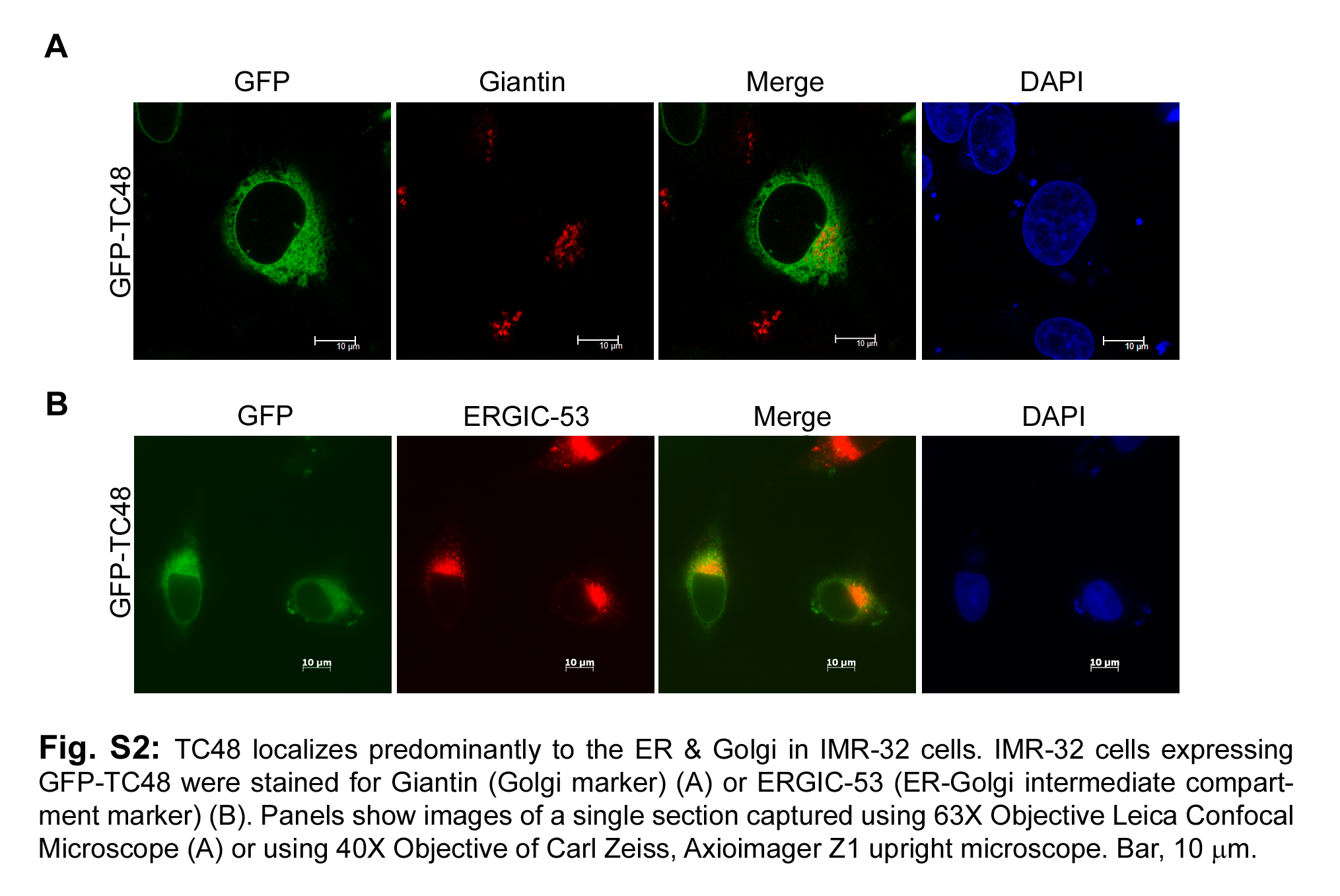

Supplement: Figure S2 — TC48 localizes predominantly to the ER & Golgi in IMR-32 cells. IMR-32 cells expressing GFP-TC48 were stained for Giantin (Golgi marker) (A) or ERGIC-53 (ER-Golgi intermediate compartment marker) (B). Panels show images of a single section captured using 63X objective of Leica Confocal Microscope (A) or using 40X Objective of Carl Zeiss, Axioimager Z1 upright microscope. Bar, 10 µm. (TIF) [file pone.0023681.s002.tif]

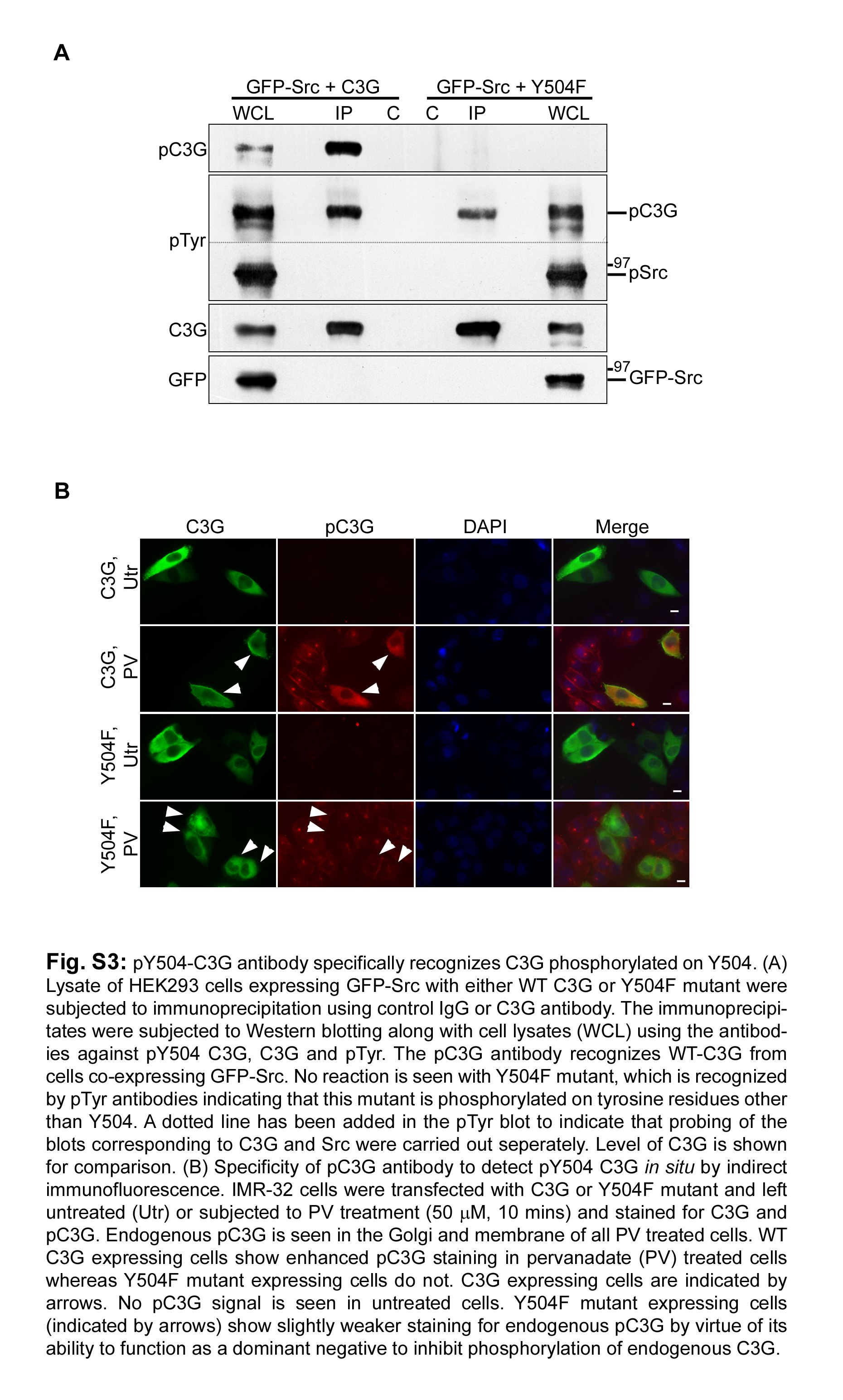

Supplement: Figure S3 — pY504-C3G antibody specifically recognizes C3G phosphorylated on Y504. (A) Lysate of HEK293 cells expressing GFP-Src with either WT C3G or Y504F mutant were subjected to immunoprecipitation using control IgG or C3G antibody. The immunoprecipitates were subjected to Western blotting along with cell lysates (WCL) using the antibodies against pY504 C3G, C3G and pTyr. The pC3G antibody recognizes WT-C3G from cells co-expressing GFP-Src. No reaction is seen with Y504F mutant, which is recognized by pTyr antibodies indicating that this mutant is phosphorylated on tyrosine residues other than Y504. A dotted line has been added in the pTyr blot to indicate that probing of the blots corresponding to C3G and Src were carried out seperately. Level of C3G is shown for comparison. (B) Specificity of pC3G antibody to detect pY504 C3G in situ by indirect immunofluorescence. IMR-32 cells were transfected with C3G or Y504F mutant and left untreated (Utr) or subjected to PV treatment (50 µM, 10 mins) and stained for C3G and pC3G. Endogenous pC3G is seen in the Golgi and membrane of all PV treated cells. WT C3G expressing cells show enhanced pC3G staining in pervanadate (PV) treated cells whereas Y504F mutant expressing cells do not. C3G and Y504F expressing cells are indicated by arrows. No pC3G signal is seen in untreated cells. Y504F mutant expressing cells show slightly weaker staining for endogenous pC3G by virtue of its ability to function as a dominant negative to inhibit phosphorylation of endogenous C3G. Bar 10 µm. (TIF) [file pone.0023681.s003.tif]

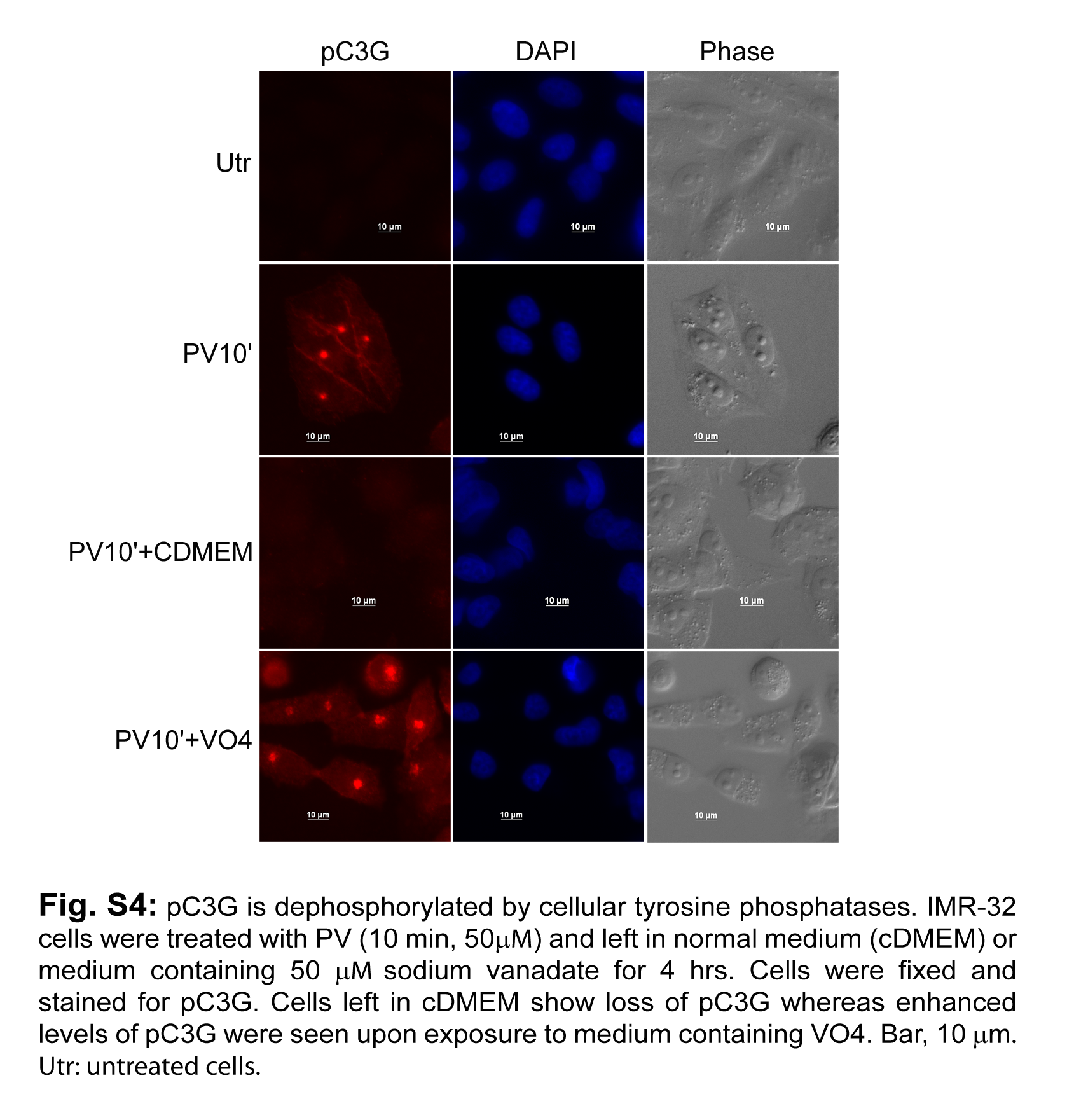

Supplement: Figure S4 — pC3G is dephosphorylated by cellular tyrosine phosphatases. IMR-32 cells were treated with PV (10 min, 50 µM) and left in normal medium (cDMEM) or medium containing 50 µM sodium vanadate for 4 hrs. Cells were fixed and stained for pC3G. Cells left in cDMEM show loss of pC3G whereas enhanced levels of pC3G were seen upon exposure to medium containing VO4. Bar, 10 µm. Utr: untreated cells. (TIF) [file pone.0023681.s004.tif]

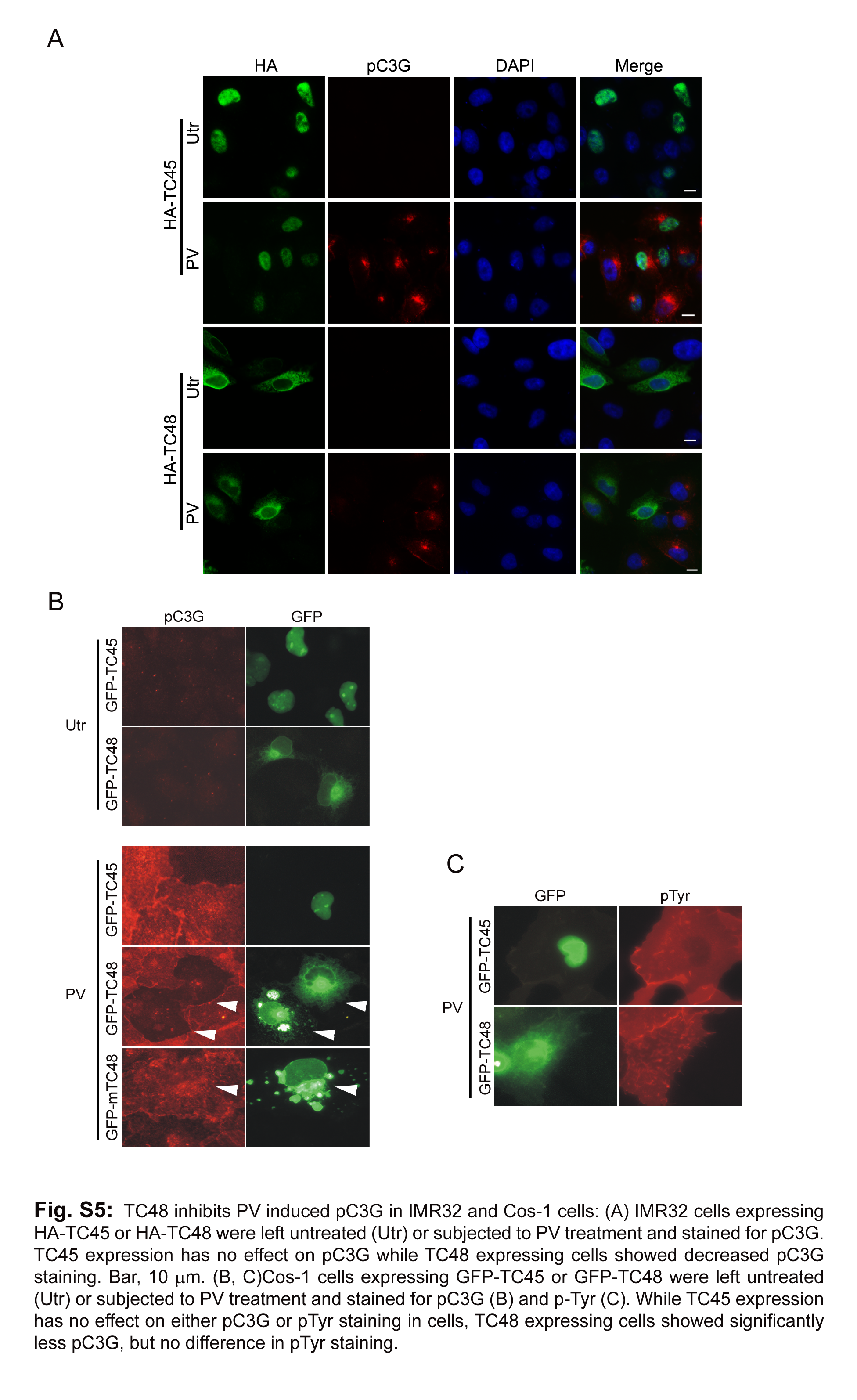

Supplement: Figure S5 — TC48 inhibits PV induced pC3G in IMR32 and Cos-1 cells: (A) IMR32 cells expressing HA-TC45 or HA-TC48 were left untreated (Utr) or subjected to PV treatment and stained for pC3G. TC45 expression has no effect on pC3G while TC48 expressing cells showed decreased pC3G staining. Bar, 10 µm. (B,C) Cos-1 cells expressing GFP-TC45 or GFP-TC48 were left untreated (Utr) or subjected to PV treatment and stained for pC3G (B) and p-Tyr (C). While TC45 expression has no effect on either pC3G or pTyr staining in cells, TC48 expressing cells showed significantly less pC3G, but no difference in pTyr staining. (TIF) [file pone.0023681.s005.tif]

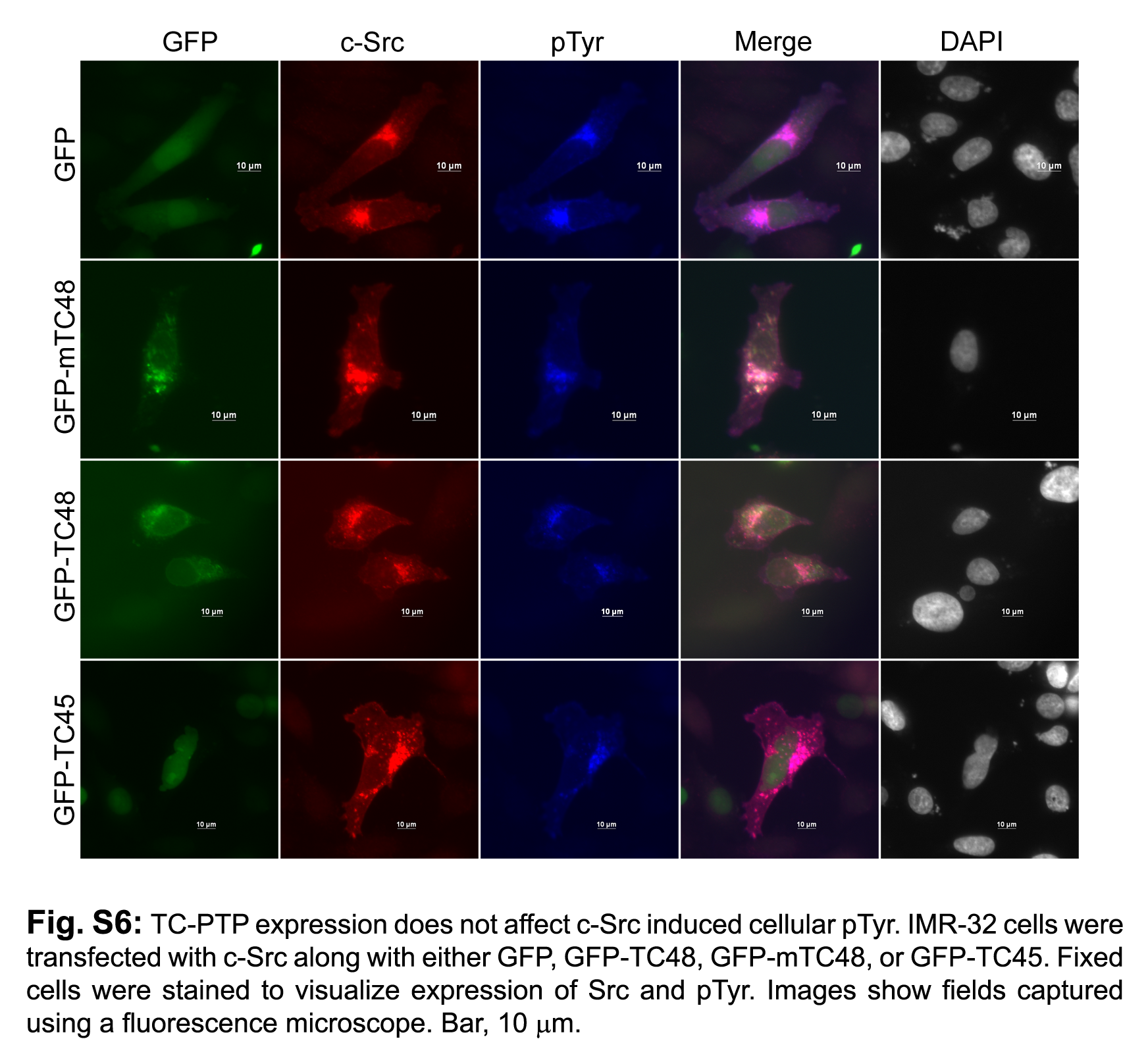

Supplement: Figure S6 — TC-PTP expression does not affect c-Src induced cellular pTyr. IMR-32 cells were transfected with c-Src along with either GFP, GFP-TC48, GFP-mTC48, or GFP-TC45. Fixed cells were stained to visualize expression of Src and pTyr. Images show fields captured using a fluorescence microscope. Bar, 10 µm. (TIF) [file pone.0023681.s006.tif]

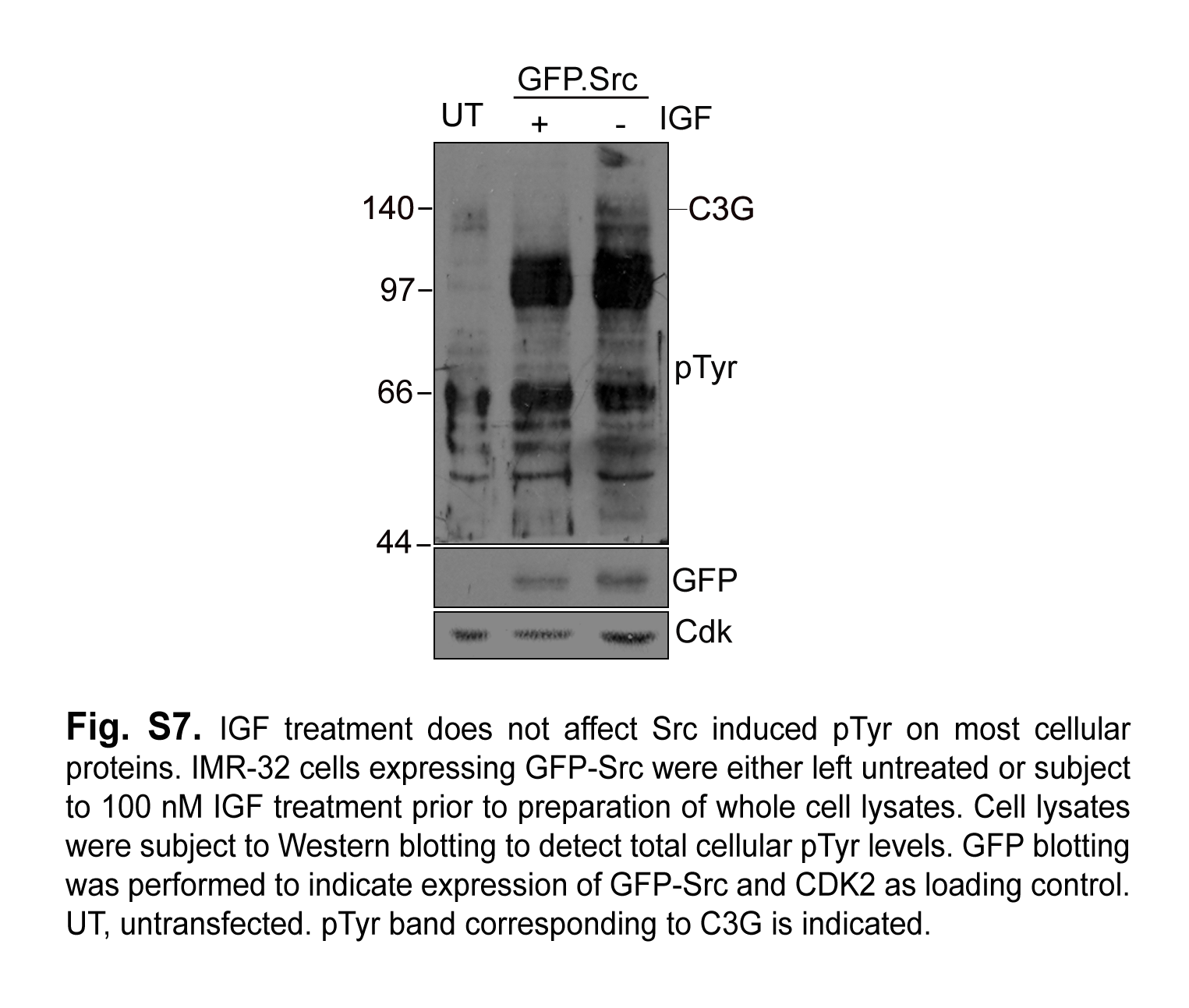

Supplement: Figure S7 — IGF treatment does not affect Src induced pTyr on most cellular proteins. IMR-32 cells expressing GFP-Src were either left untreated or subject to 100 nM IGF treatment prior to preparation of whole cell lysates. Cell lysates were subject to Western blotting to detect total cellular pTyr levels. GFP blotting was performed to indicate expression of GFP-Src and CDK2 as loading control. UT, untransfected. pTyr band corresponding to C3G is indicated. (TIF) [file pone.0023681.s007.tif]
